# Supplementary material for: A Delphi Study to Determine International and National Equestrian Expert Opinions on Domains and Sub-Domains Essential to Managing Sporthorse Health and Welfare in the Olympic Disciplines
Source: Animals (Basel). 2023 Nov 2;13(21):3404. doi: 10.3390/ani13213404 (PMC10650931; doi:10.3390/ani13213404)
Supplement: Supplementary file 1 [file animals-13-03404-s001.zip › animals-2646666-supplementary.pdf]

Supplementary File S1: Monitoring Sporthorse Health and Welfare

Key to analysis for essential rating to sporthorse health and welfare:

Above 70% agreement Consensus agreed	Above average agreement	Below average agreement No consensus

Average agreement:

Above 90%	80-89%	70-79%	60-69%	50-59%	Less than 50%
Excellent agreement	Good agreement	Acceptable agreement	Moderate agreement	Poor agreement	Lack of agreement

General Management: Factors which should be regularly assessed

Table S1: Overview of agreement: General Management

**Average agreement: 48%**	**Agreement: Essential to sporthorse health and welfare**	**Agreement: Can currently be assessed accurately**
Environment and climate management e.g., air quality, temperature, and humidity	54%	11%
Behavioural assessment of the horse-human interaction when ridden	66%	14%
Workload details e.g., duration, frequency, intensity, type of exercise	76%	69%
Travelling schedule e.g., frequency, duration, hydration, feeding etc	78%	17%
Surface management	70%	43%
Horse fitness for exercise	90%	58%
Horse recovery after exercise	72%	44%
Musculoskeletal health e.g., gait and lameness assessment	78%	22%
Behavioural assessment: horse	79%	19%
Behavioural assessment: handler / rider	56%	17%
Behavioural assessment: horse and rider combination	60%	26%
Behavioural assessment of the horse-human interaction: In-hand	56%	3%
Appropriate use of tack and equipment	84%	56%
Biomarkers of disease	34%	9%
Blood profiles for disease and injury	26%	9%
Injury records e.g., type of injury, days out of training, recurrence etc	78%	23%
Choice and appropriate fit of tack and equipment	72%	51%

Husbandry monitoring e.g., stable size, turnout, sleep etc	60%	3%
Nutrition e.g., forage, concentrates, water, use of supplements etc	70%	3%
Blood profiling: red blood cell counts	2%	11%
Blood profiling: white blood cell counts	6%	9%
Blood profiling: total blood cell counts	2%	12%
Health related biomarkers	42%	6%
Biomarkers of inflammation	46%	9%
Health assessment e.g., temperature, pulse, respiration etc	68%	17%
Adherence to rules and regulations e.g., FEI and National Governing Body	70%	11%
Blood profiles for disease and injury	32%	17%
Farriery / hoof care e.g., frequency, changes to routine etc.	82%	6%
Medication records	68%	15%
Record of any supplements used	32%	17%
Body weight (using a weigh bridge)	14%	14%
Body Condition Score	44%	11%
Pain scoring of unridden horse	46%	14%
Pain scoring of ridden horse	58%	6%
Welfare assessment e.g., using an established welfare tool	66%	11%

Key: Positive values 1 to 100% represent agreement for if the area is considered essential; 0 represents no clear agreement if area is essential or not; negative values -1 to -100% indicate disagreement for is the area is considered not essential.

Climate and Environment Monitoring

Table S2: Overview of agreement: Climate and Environment Monitoring

**Average agreement: 48%**	**Agreement: Essential to sporthorse health and welfare**	**Agreement: Can currently be assessed accurately**
Quality of surfaces used for ridden exercise	80%	55%
Internal air quality: housing / indoor arenas	68%	57%
Humidity: housing / indoor arenas	65%	70%
Internal humidity: housing / indoor arenas	59%	72%
Moisture content of surfaces	57%	45%
Quality of surfaces used for turnout	48%	43%
Quality of surfaces used for non-ridden exercise	42%	49%
External temperature	42%	74%
Environmental humidity	34%	68%
Direct / indirect sunlight	22%	52%
External air quality	16%	50%
Wind direction and speed	10%	68%

Key: Positive values 1 to 100% represent agreement for if the area is considered essential; 0 represents no clear agreement if area is essential or not; negative values -1 to -100% indicate disagreement for is the area is considered not essential.

Stable Management Monitoring

Table S3: Overview of agreement: Stable Management Monitoring

**Average agreement: 56%**	**Agreement: Essential to sporthorse health and welfare:**	**Agreement: Can currently be assessed accurately:**
Quantity and quality of bedding available for use	100%	61%
Monitoring of stable size to horse size	100%	71%
Frequency, quantity and type of feed and water provided	100%	74%
Observation and recording of handler / rider / horse interaction	100%	48%
Measurement and recording of exercise type, frequency and duration outside…	82%	58%
Regular veterinary examinations: general health	80%	73%
Observation and recording of horse behaviour	78%	42%
Pain assessment	70%	45%
Regular veterinary examinations: lameness	68%	68%
Assessment of ventilation	63%	65%
Monitoring of tack and equipment used for handling	55%	52%
Measurement and recording of time spent stabled	49%	58%
Analysis of temperature in stables	48%	71%
Welfare assessment e.g. using an established welfare tool	43%	27%
Measurement and recording of time spent outside the stable	43%	58%
Analysis of air quality in stables	40%	61%
Observation and recording of type and frequency of social interaction between	34%	50%
Analysis of humidity in stables	32%	71%
Use of behavioural assessment tools such as Equifacs	26%	27%
Analysis of direct/ indirect sunlight	12%	42%
Testing measures of stress	12%	19%
Use of wearable technology to assess horse behaviour	0%	23%
Video analysis within stable environment	-25%	33%

Key: Positive values 1 to 100% represent agreement for if the area is considered essential; 0 represent no clear agreement if area is essential or not; negative values -1 to -100% indicate disagreement for is the area is considered not essential.

Health and Veterinary Assessment and Monitoring

Table S4: Overview of agreement: Health and Veterinary Assessment and Monitoring

**Average agreement: 54%**	**Agreement: Essential to sporthorse health and welfare:**	**Agreement: Can currently be assessed accurately:**
Gait analysis: visual lameness assessment	100%	52%
Lameness records	94%	58%
Assessment of presence of pain	86%	35%
Clinical health check (e.g. temperature, heart rate, respiratory rate, head…	80%	65%
Disease and injury records	76%	55%
Vaccination records	74%	71%
Records of medication	72%	47%
Faecal analysis e.g. for parasites	60%	71%
Assessment of muscle symmetry	56%	39%
Records of veterinary interventions and treatments	56%	52%
Feed analysis	56%	48%
Behavioural assessment of pain (e.g. ridden horse ethogram, grimace scale)	54%	42%
Colic records	54%	63%
Monitoring of hydration status	54%	39%
Body weight monitoring	48%	55%
Body condition score monitoring	40%	58%
Gait analysis using specifically designed technology	40%	48%
Heart rate and/or ECG analysis	30%	65%
Respiratory health monitoring e.g., scoping	14%	58%
Routine blood analysis (e.g., biochemistry and haematology)	4%	74%

Key: Positive values 1 to 100% represent agreement for if the area is considered essential; 0 represent no clear agreement if area is essential or not; negative values -1 to -100% indicate disagreement for is the area is considered not essential.

Gait / Lameness Assessment Monitoring

Table S5: Overview of agreement: Gait / Lameness Assessment Monitoring

**Average agreement: 30%**	**Agreement: Essential to sporthorse health and welfare:**	**Agreement: Can currently be assessed accurately:**
Visual assessment of lameness	86%	52%
Veterinary assessment of gait / lameness	80%	58%
Behavioural / pain assessment	74%	39%
Injury history	60%	58%
Asymmetry analysis	56%	45%
Stride duration, length and frequency analysis	42%	42%
Joint movement analysis	42%	45%
Computer based motion analysis	16%	52%
Commercial gait analysis systems designed to be used by owners / riders	4%	29%
Use of video footage to assess gait	4%	32%
Saddle pressure mats	-4%	29%
Force or pressure plate analysis	-28%	32%
Thermography	-42%	26%

Key: Positive values 1 to 100% represent agreement for if the area is considered essential; 0 represent no clear agreement if area is essential or not; negative values -1 to -100% indicate disagreement for if the area is considered not essential.

Behaviour Assessment and Monitoring

Table S6: Overview of agreement: Behaviour Assessment and Monitoring

**Average agreement: 28%**	**Agreement: Essential to sporthorse health and welfare:**	**Agreement: Can currently be assessed accurately:**
Observation of behaviour	74%	35%
Pain assessment: ridden	62%	29%
Monitoring of the fit and frequency of use of tack and equipment	60%	35%
Pain assessment: non-ridden	54%	30%
Behavioural assessment tools	36%	26%
Records of daily routine including social interaction, feeding and exercise	34%	52%
Animal Welfare Indicators (AWIN) protocol	32%	26%
Ridden horse ethogram	28%	37%
Ethograms	18%	39%
Heart rate analysis (at rest)	4%	68%
Cortisol assessment	2%	53%
Biomarkers of stress	-2%	29%
Video analysis	-4%	45%
Heart rate variability analysis (at rest)	-4%	65%

Key: Positive values 1 to 100% represent agreement for if the area is considered essential; 0 represent no clear agreement if area is essential or not; negative values -1 to -100% indicate disagreement for if the area is considered not essential.

Fitness / Recovery Assessment and Monitoring

Table S7: Overview of agreement: Fitness / Recovery Assessment and Monitoring

**Average agreement: 41%**	**Agreement: Essential to sporthorse health and welfare:**	**Agreement: Can currently be assessed accurately:**
Horse fitness for exercise	90%	65%
Gait analysis: visual lameness assessment	74%	52%
Horse recovery after exercise	72%	68%
Clinical health check (e.g. temperature, pulse, respiratory rate, head to tail)	66%	68%
Behavioural assessment of pain (e.g. ridden horse ethogram, grimace scale)	60%	39%
Workload assessment	58%	30%
Temperature: rectal, skin or core temperature	56%	71%
Behavioural assessment of ridden work	54%	43%
Hydration status	54%	55%
Heart rate monitoring during recovery	52%	74%

Blood profiling: lactate levels	52%	61%
Visual assessment of muscle symmetry	48%	48%
Heart rate monitoring during exercise	40%	61%
Gait analysis using specifically designed technology	34%	45%
Standardised exercise test	26%	37%
Blood profiling: electrolyte analysis	24%	61%
Assessment of presence of muscular pain (e.g. handheld algometer)	22%	35%
Health related biomarkers	16%	48%
Blood profiling: haematology (e.g. white and red blood cell counts)	14%	65%
Blood profiling: biochemistry (e.g. biomarkers of health, inflammation and...	12%	65%
Heart rate variability at rest	6%	68%
Salivary cortisol assessment	-26%	39%

Key: Positive values 1 to 100% represent agreement for if the area is considered essential; 0 represent no clear agreement if area is essential or not; negative values -1 to -100% indicate disagreement for if the area is considered not essential.

Training Assessment and Monitoring

Table S8: Overview of agreement: Training Assessment and Monitoring

**Average agreement: 50%**	**Agreement: Essential to sporthorse health and welfare:**	**Agreement: Can currently be assessed accurately:**
Long term monitoring of training	94%	59%
Record of feed, forage, and water	94%	69%
Time spent stabled	82%	59%
Workload monitoring: duration, activity: type and intensity and frequency	80%	57%
Gait analysis: visual lameness assessment by owner, groom or rider	80%	40%
Warm up monitoring: duration, activity: type and intensity and frequency	80%	67%
Food and water intake and frequency of feeding	74%	59%
Opportunities and time spent in free exercise (not related to training)	74%	57%
Veterinary assessment of gait / lameness	72%	62%
Injury and disease records	68%	66%
Clinical health check (e.g. temperature, heart rate, respiratory rate, head...	68%	63%
Fitness testing of the rider	68%	59%
Medication records	62%	69%

Assessment of presence of muscular pain (e.g. handheld manometer)	60%	27%
Behavioural assessment of ridden work	60%	33%
Assessment of muscle symmetry	48%	30%
Behavioural assessment of pain (e.g. ridden horse ethogram, grimace scale)	48%	40%
Temperature: body, surface and rectal	48%	66%
Records of days lost from training	48%	69%
Records of supplements used	48%	69%
Saddle pressure (magnitude and distribution)	46%	43%
Opportunities for social interaction	46%	45%
Rein tension evaluation (magnitude and symmetry)	42%	50%
Sleep quantity and quality	42%	28%
Standardised exercise tests to assess fitness level	34%	50%
Hydration status	34%	59%
Body condition score monitoring	28%	57%
Bodyweight monitoring (using weight bridge)	24%	60%
Gait analysis using specifically designed technology	20%	53%
Heart rate monitoring	18%	62%
Bloods: Lactate	18%	67%
Bloods: biochemistry (e.g., biomarkers of inflammation and muscle damage)	14%	70%
Bloods: haematology (e.g., white and red blood cell counts)	14%	70%
Bloods: electrolyte analysis	14%	70%
Bloods: cortisol analysis	-14%	57%

Key: Positive values 1 to 100% represent agreement for if the area is considered essential; 0 represent no clear agreement if area is essential or not; negative values -1 to -100% indicate disagreement for if the area is considered not essential.

Monitoring in Competition

Table S9: Overview of agreement: Monitoring in Competition

**Average agreement: 44%**	**Agreement: Essential to sporthorse health and welfare:**	**Agreement: Can currently be assessed accurately:**
Veterinary assessment of gait / lameness	94%	67%
Medication records	88%	76%
Gait analysis: visual lameness assessment	86%	63%
Warm up monitoring: duration, activity: type and intensity and frequency	80%	57%
Clinical health check (e.g. temperature, heart rate, respiratory rate, head...	80%	70%

Workload monitoring: duration, activity: type and intensity and frequency	80%	60%
Records of competition undertaken: no of classes, duration, performance etc	74%	77%
Food and water intake and frequency of feeding	74%	50%
Records of travelling: distance, time, watering, nutrition	72%	69%
Records of supplements used	68%	46%
Injury and disease records	66%	73%
Sleep quantity and quality	54%	67%
Opportunities for social interaction	54%	40%
Time spent stabled	54%	73%
Behavioural assessment of pain – stabled (e.g. ridden horse ethogram, grimace scale)	52%	47%
Opportunities and time spent in free exercise	52%	52%
Monitoring of recovery after exercise	46%	70%
Access to long term training records	46%	60%
Monitoring and tracking of performance in horse and rider combinations	46%	55%
Stable size measurement	46%	73%
Daily veterinary assessment	46%	67%
Monitoring of adherence to competition / governing body rules	40%	50%
Records of days lost from training	40%	72%
Access to long term competition records	34%	69%
Heart rate monitoring	32%	73%
Measures of stress	28%	27%
Gait analysis using specifically designed technology	20%	53%
Saddle pressure magnitude and distribution	14%	43%
Rein tension evaluation	14%	45%
Video analysis and records of human horse interactions	6%	70%
Bloods profiling: lactate	-6%	77%
Blood profiling to assess health	-6%	57%
Health related biomarkers	-32%	50%
Blood or saliva cortisol measurement	-38%	50%

Key: Positive values 1 to 100% represent agreement for if the area is considered essential; 0 represent no clear agreement if area is essential or not; negative values -1 to -100% indicate disagreement that the area is considered not essential.

Welfare Assessment and Monitoring

Table S10: Overview of agreement: Welfare Assessment and Monitoring

**Average agreement: 49%**	**Agreement: Essential to sporthorse health and welfare:**	**Agreement: Can currently be assessed accurately:**
Pain assessment	88%	41%
Regular records of horse health	84%	66%
Regular records of horse management	80%	64%
Tack and equipment use and fit assessment	72%	41%
Type, presence, severity and frequency of stereotypic behaviours	52%	48%
Biomarkers of stress	44%	29%
Measures of stress e.g. cortisol	44%	52%
Use of validated welfare tools	40%	24%
Animal Welfare Indicators (AWIN) protocol	2%	21%
Video analysis of horse human interactions	-18%	21%

Key: Positive values 1 to 100% represent agreement for if the area is considered essential; 0 represent no clear agreement if area is essential or not; negative values -1 to -100% indicate disagreement for if the area is considered not essential.

Frequency of monitoring

Table S11: Expert opinion for how often measures related to sport horse health and welfare should be monitored; shaded squares indicate the most frequent response.

	More than once per day	Daily	Weekly	3-5 times per week	Monthly	Ad hoc
Environment and climate management e.g., air quality, temperature, and humidity	19%	31%	22%	8%	8%	11%
Behavioural assessment of the horse-human interaction when ridden	3%	39%	19%	6%	19%	14%
Workload details e.g., duration, frequency, intensity, type of exercise	3%	36%	39%	3%	14%	6%
Travelling schedule e.g., frequency, duration, hydration, feeding etc.	11%	14%	17%	8%	31%	19%
Surface management	6%	56%	19%	8%	6%	6%
Horse fitness for exercise	0%	56%	25%	8%	8%	3%
Horse recovery after exercise	3%	75%	8%	0%	6%	8%
Musculoskeletal health e.g., gait and lameness assessment	0%	56%	17%	3%	14%	11%
Behavioural assessment: horse	6%	37%	17%	11%	17%	11%
Behavioural assessment: handler / rider	3%	31%	17%	11%	17%	20%
Behavioural assessment: horse and rider combination	3%	37%	20%	6%	20%	14%

Behavioural assessment of the horse-human interaction: In-hand	3%	39%	17%	8%	17%	17%
Appropriate use of tack and equipment	0%	53%	14%	3%	19%	11%
Biomarkers of disease	0%	3%	3%	3%	23%	69%
Blood profiles for disease and injury	0%	0%	6%	3%	29%	63%
Injury records e.g., type of injury, days out of training, recurrence etc	0%	18%	6%	3%	50%	24%
Choice and appropriate fit of tack and equipment	0%	44%	11%	3%	25%	17%
Husbandry monitoring e.g., stable size, turnout, sleep etc	0%	17%	36%	8%	17%	22%
Nutrition e.g., forage, concentrates, water, use of supplements etc	3%	36%	19%	3%	33%	6%
Blood profiling: red blood cell counts	0%	3%	3%	3%	31%	61%
Blood profiling: white blood cell counts	0%	3%	3%	3%	25%	67%
Blood profiling: total blood cell counts	0%	3%	3%	3%	26%	66%
Health related biomarkers	0%	6%	3%	3%	29%	60%
Biomarkers of inflammation	0%	3%	3%	0%	26%	69%
Health assessment e.g., temperature, pulse, respiration etc	16%	41%	14%	8%	3%	19%
Adherence to rules and regulations e.g., FEI and National Governing Body	0%	19%	14%	3%	24%	41%
Farriery / hoof care e.g., frequency, changes etc	0%	16%	16%	5%	54%	8%

Medication records	0%	32%	11%	3%	24%	30%
Record of any supplements used	0%	22%	22%	3%	25%	28%
Body weight (using a weigh bridge)	0%	5%	30%	5%	43%	16%
Body Condition Score	0%	3%	27%	8%	51%	11%
Pain scoring of unridden horse	0%	17%	19%	8%	25%	31%
Pain scoring of ridden horse	0%	19%	16%	11%	32%	22%
Welfare assessment e.g., using an established welfare tool	0%	11%	19%	14%	30%	27%

Other areas experts commented as important in free text: Daily check by groom; Monthly quantitative gait assessment; keeping a record / logbook of key elements of horse management such as feed and exercise.
